# On Poisons in Relation to Medical Jurisprudence and Medicine

**Published:** 1859-09

**Authors:** 


ART. VI.—*On Poisons in Relation to Medical Jurisprudence and Medicine.* By ALFRED SWAINE TAYLOR, M.D., F.R.S., etc. Second American, from the second and revised London edition. 8vo., pp. 755. Philadelphia: Blanchard & Lea. 1859.

TEN years have elapsed since the first appearance of this work, during which time the author, probably the leading toxicologist in England, has been almost incessantly occupied in the investigations of poison in criminal cases, the results of which have, from time to time, appeared in the London periodicals. All that is of importance in the great mass of these inquiries will be found to be comprised in the present edition, which has been completely changed in its arrangement. Taking the same position as his treatise on "Medical Jurisprudence," it can safely be said to be the most reliable work on the subjects of which it treats that has as yet appeared in the English language.

